# Clinical and Pulmonary CT Characteristics of Patients Infected With the SARS-CoV-2 Omicron Variant Compared With Those of Patients Infected With the Alpha Viral Strain

**DOI:** 10.3389/fpubh.2022.931480

**Published:** 2022-07-12

**Authors:** Naibin Yang, Chuwen Wang, Jiajia Huang, Jing Dong, Jihui Ye, Yuan Fu, Jingfeng Huang, Daojie Xu, Gang Cao, Guoqing Qian

**Affiliations:** ^1^Department of Infectious Diseases, Ningbo First Hospital, Ningbo University, Ningbo, China; ^2^School of Medicine, Ningbo University, Ningbo, China; ^3^Department of Emergency, Ningbo First Hospital, Ningbo University, Ningbo, China; ^4^Department of Intensive Care Unit, Ningbo First Hospital, Ningbo University, Ningbo, China; ^5^Department of Radiology, Ningbo First Hospital, Ningbo University, Ningbo, China; ^6^Department of Anesthesiology, Huashan Hospital, Fudan University, Shanghai, China; ^7^Department of Anesthesiology and Pain Medical Center, Ningbo First Hospital, Ningbo University, Ningbo, China

**Keywords:** COVID-19, SARS-CoV-2, Omicron, Alpha, computed tomography

## Abstract

**Background:**

Omicron has become the dominant variant of severe acute respiratory syndrome coronavirus 2 (SARS-CoV-2) globally. We aimed to compare the clinical and pulmonary computed tomography (CT) characteristics of the patients infected with SARS-CoV-2 Omicron with those of patients infected with the Alpha viral strain.

**Methods:**

Clinical profiles and pulmonary CT images of 420 patients diagnosed with coronavirus disease-2019 (COVID-19) at Ningbo First Hospital between January 2020 and April 2022 were collected. Demographic characteristics, symptoms, and imaging manifestations of patients infected with the SARS-CoV-2 Omicron variant were compared with those of patients infected with the Alpha strain.

**Results:**

A total of 38 patients were diagnosed to be infected with the Alpha strain of SARS-CoV-2, whereas 382 patients were thought to be infected with the Omicron variant. Compared with patients infected with the Alpha strain, those infected with the Omicron variant were younger, and a higher proportion of men were infected (*P* < 0.001). Notably, 93 (24.3%) of the patients infected with Omicron were asymptomatic, whereas only two (5.3%) of the patients infected with the Alpha strain were asymptomatic. Fever (65.8%), cough (63.2%), shortness of breath (21.1%), and diarrhea (21.1%) were more common in patients infected with the SARS-CoV-2 Alpha strain, while runny nose (24.1%), sore throat (31.9%), body aches (13.6%), and headache (12.3%) were more common in patients with the Omicron variant. Compared with 33 (86.84%) of 38 patients infected with the Alpha strain, who had viral pneumonia on pulmonary CT images, only 5 (1.3%) of 382 patients infected with the Omicron variant had mild foci. In addition, the distribution of opacities in the five patients was unilateral and centrilobular, whereas most patients infected with the Alpha strain had bilateral involvement and multiple lesions in the peripheral zones of the lung.

**Conclusion:**

The SARS-CoV-2 Alpha strain mainly affects the lungs, while Omicron is confined to the upper respiratory tract in patients with COVID-19.

## Introduction

In December 2019, cases of pneumonia of unknown cause were identified in Wuhan, Hubei Province, China. It was caused by the seventh member of the coronavirus family, which was subsequently named severe acute respiratory syndrome coronavirus 2 (SARS-CoV-2) (1). The new virus caused pneumonia, and the infection was named coronavirus disease-2019 (COVID-19). COVID-19 has spread rapidly worldwide owing to its highly contagious characteristics in the past 2 years (2), leading to a sustained pandemic. It is mainly attributed to the serial emergence of the new genetic variants of SARS-CoV-2 (3) and difficulties in the distribution of highly effective vaccines (4). The World Health Organization has successively designated and recognized five SARS-CoV-2 variants of concern as follows: Alpha (B.1.1.7 and descendant lineages), Beta (B.1.351), Gamma (P.1), Delta (B.1.617.2 and AY lineages), and Omicron (B.1.1.529 and BA lineages) (5). Omicron was initially reported in November 2021 in Botswana and South Africa (6, 7) and has quickly become the dominant variant worldwide.

“Cold-like” symptoms, such as runny nose, headache, and fatigue, are the most common symptoms in patients infected with the Omicron variant (8–10). Omicron was reported in November 2021 in a quarantined hotel in Hong Kong, China (11). Patients with COVID-19 infected with the Omicron variant have continuously been reported in China (12–14). Currently, as of April 24, in Shanghai, China, the Omicron variant has caused a large number of SARS-CoV-2 infections, and COVID-19 has caused 87 deaths. Some of these patients have been transferred to surrounding cities, including Ningbo City, for quarantine and treatment. Because of the great efforts of Shanghai and surrounding cities, the current omicron wave of the COVID-19 pandemic was gradually under control. Less was known about the clinical characteristics and pulmonary computed tomography (CT) features of Chinese patients infected with the Omicron variant, particularly compared with patients infected with the Alpha strain. In this study, we compared the clinical profiles and pulmonary CT of patients with COVID-19 that was caused by the SARS-CoV-2 Omicron variant and Alpha strain.

## Methods

We conducted a retrospective study using data from all patients with COVID-19 admitted to Ningbo First Hospital or a Ningbo First Hospital-administered shelter hospital between January 2020 and April 2022. This study was approved by the Medical Ethics Committee of Ningbo First Hospital (approval number: 2020-R052; 2022RS065). The requirement for written informed consent from the patients was waived due to the retrospective study design and no additional intervention or potential harm.

Coronavirus disease-2019 was diagnosed based on a positive result for SARS-CoV-2 RNA using samples from throat or nasal swabs (15, 16). Patients with COVID-19 were classified into two groups, namely, those infected with the Alpha variant and those infected with the Omicron variant. The patients were classified according to the predominant viral variant circulating in China at the time of their hospital admission. Urgent viral whole-genome sequencing was unavailable at the time of the outbreak. Demographic characteristics, clinical symptoms, and pulmonary CT images at admission were systematically collected from electronic medical records and reviewed by two experienced physicians (N. Yang & G. Qian). All patients underwent pulmonary CT scans before admission or within 24 h after admission except 8 patients with the Omicron variant including 7 patients aged lower than 14 years old and one pregnant. Two experienced radiologists (Y. Fu & J. Huang) with more than 5 years of experience reviewed all CT images. Image characteristics included the distribution of lesions and the ground glass opacity (GGO) in the lungs. The affected lungs were counted.

Age was expressed as median (interquartile range) because it was not normally distributed; a comparison between the two groups was performed using the Mann–Whitney *U*-test. Categorical variables were presented as counts and percentages, and chi-square tests or Fisher's exact tests were used to test the significance between the two groups. A value of *P* < 0.05 (two-sided) was considered statistically significant. All statistical analyses were performed using the IBM SPSS statistics version 26.0 (IBM Corp, Armonk, NY, USA) and the EmpowerStats software (X&Y Solutions, Inc., Boston, MA, USA).

## Results

### Demographic and Clinical Characteristics of the Patients

Of the total number of patients, 38 patients were presented at the hospital when the Alpha variant was dominant and 382 patients were presented when the Omicron variant was dominant. Patients infected with Omicron were predominantly younger than 40 years of age (76.2%; average age, 32 years) compared with patients infected with the Alpha strain who tended to be older than 40 years (84.2%; average age, 57 years). Compared with patients infected with the Alpha strain, the proportion of men was higher (41.9%) in patients infected with Omicron (*P* < 0.001).

The clinical characteristics of patients infected with the Alpha strain and the Omicron variant were seemly different. Two (5.3%) patients infected with the Alpha strain were asymptomatic. Fever (65.8%), cough (63.2%), shortness of breath (21.1%), and diarrhea (21.1%) were more common in patients infected with the Alpha strain. Notably, 93 (24.3%) of the patients infected with Omicron were asymptomatic. Runny nose (24.1%), sore throat (31.9%), body aches (13.6%), and headache (12.3%) were more common in patients with the Omicron variant. The results are shown in Table 1.

**Table 1:** Demographic and clinical characteristics in patients infected with Omicron variant and the Alpha strain.

	**Omicron (*n* = 382)**	**Alpha (*n* = 38)**	***P*-value**
Age (years)	32 (26–38)	57 (52–63)	<0.001
**Age groups:** ***N*** **(%)**
<18	9 (2.4%)	1 (2.6%)	<0.001
18–39	291 (76.2%)	5 (13.2%)	
40–59	66 (17.3%)	19 (50.0%)	
≥60	16 (4.2%)	13 (34.2%)	
Men: *N* (%)	160 (41.9%)	11 (28.9%)	0.122
**Symptoms:** ***N*** **(%)**
Asymptomatic	93 (24.3%)	2 (5.3%)	0.007
Running nose	92 (24.1%)	3(7.9%)	<0.001
Headache	47 (12.3%)	4 (10.5%)	0.749
Fatigue	71 (18.6%)	16 (43.2%)	<0.001
Sore throat	122 (31.9%)	7 (18.4%)	0.006
Body pain	52 (13.6%)	2 (5.3%)	0.155
Fever	154 (40.3%)	25 (65.8%)	0.002
Cough	157 (41.1%)	24 (63.2%)	0.009
Expectoration	111 (29.1%)	11 (28.9%)	0.989
Shortness of breath	10 (2.6%)	8 (21.1%)	<0.001
Diarrhea	34 (8.9%)	8 (21.1%)	0.017

### Comparison of the Pulmonary CT Features in the Patients

It is noted that 33 (86.84%) of 38 patients infected with the Alpha strain had viral pneumonia on pulmonary CT images, while only five (1.3%) of 382 patients with the Omicron variant showed mild foci. Also, 31 (93.9%) of 33 patients with viral pneumonia infected with the Alpha strain showed bilateral involvement, and only two (6.1%) patients showed unilateral involvement. However, all five patients with viral pneumonia infected with the Omicron variant showed bilateral involvement. The results are shown in Table 2.

**Table 2:** Comparison of pulmonary CT features in patients infected with Omicron variant and the Alpha strain.

	**Omicron (*n* = 374)**	**Alpha (*n* = 38)**	***P*-value**
**Viral pneumonia:** ***N*** **(%)**	5/374 (1.3%)	33 (86.84%)	<0.001
**Distribution of opacities:** ***N*** **(%)**
Unilateral	5 (100%)	2 (6.1%)	<0.001
Bilateral	0 (0%)	31 (93.9%)	<0.001
**Lobes involvement:** ***N*** **(%)**
Left upper lobe	0 (0%)	22 (66.7%)	<0.001
Left lower lobe	1 (20%)	22 (66.7%)	<0.001
Right upper lobe	1 (20%)	17 (51.5%)	<0.001
Right middle lobe	2 (40%)	9 (2.8%)	<0.001
Right lower lobe	2 (40%)	32 (97%)	<0.001

**Figure 1:**
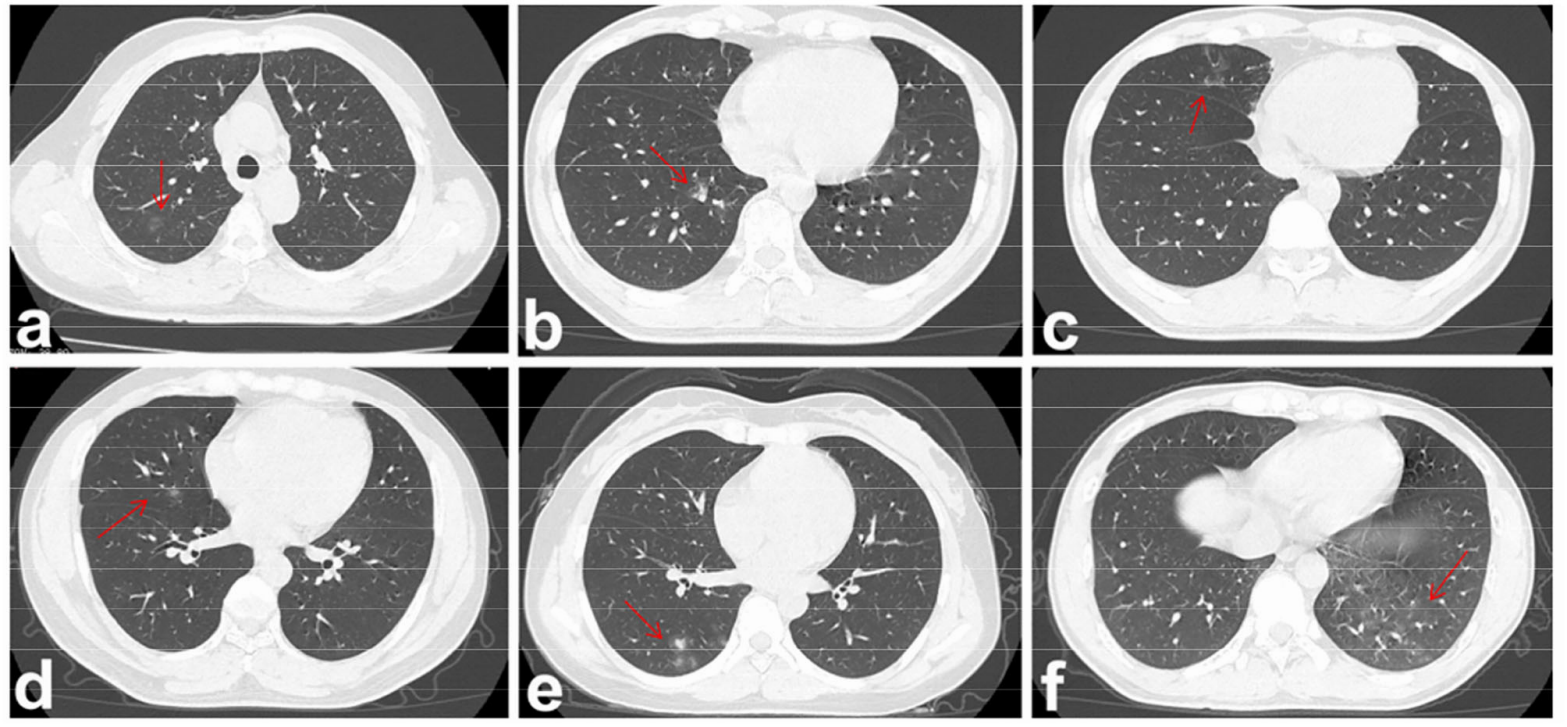
Viral pneumonia of pulmonary CT imaging in the five patients infected with Omicron variant. The lesions were marked by red arrows in panels **(a–f)**. **(a)** Patient 1: GGO in the right upper lobe of a 38-year-old woman. She presented with fever and sore throat. **(b)** and **(c)** Patient 2: GGO in the right lower and middle lobe of a 27-year-old man, respectively. He presented only with low degree of fever. **(d)** Patient 3: GGO in the right middle lobe of a 28-year-old man. He was asymptomatic. **(e)** Patient 4: GGO in the right lower lobe of a 28-year-old woman. She presented with body pain and fatigue. **(f)** Patient 5: GGO in the left lower lobe of a 29-year-old man. He presented with fever and sore throat.

**Figure 2:**
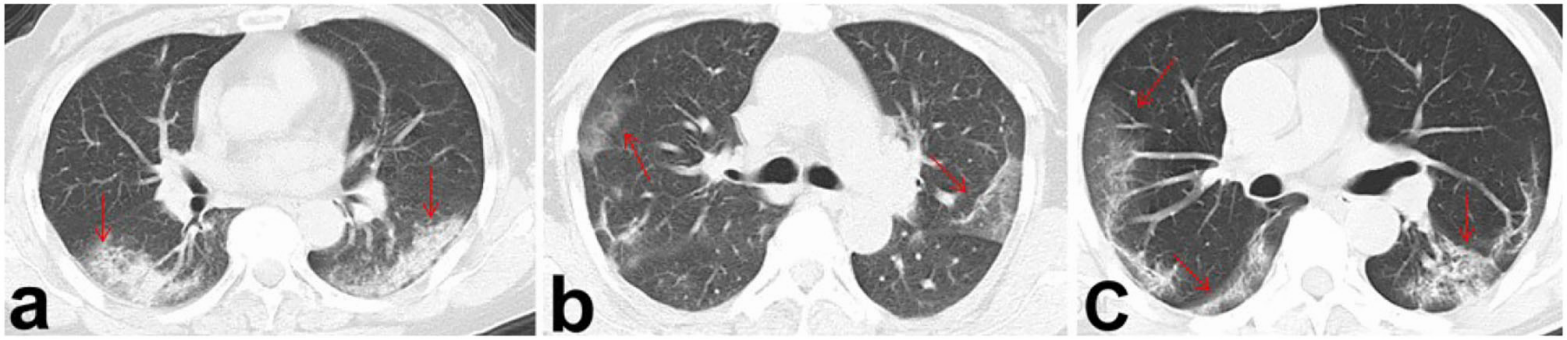
Viral pneumonia of pulmonary CT imaging in three patients infected with the Alpha strain. The lesions were marked by red arrows in panels **(a–c)**. Bilateral involvement and multiple lesions located in peripheral zones of the lungs were found.

In addition, the distribution of opacities in patients infected with the Omicron variant was unilateral and centrilobular (Figure 1), whereas most patients infected with the Alpha strain had bilateral involvement and multiple lesions in the peripheral zones of the lung (Figure 2).

In five patients infected with Omicron, the left lung was rarely involved, and only one had a mild distribution of opacities in the left lower lobe. Involvement of the right upper lobe, right middle lobe, and right lower lobe was noted in one, two, and two patients, respectively. In addition, only one patient's both lobes were involved, whereas the other patients had unilateral and single-lobe involvement. The results are shown in Table 3.

**Table 3:** Lobe involvement of viral pneumonia in patients infected with Omicron.

**Lobe involvement**	**Patient 1**	**Patient 2**	**Patient 3**	**Patient 4**	**Patient 5**
Left upper lobe	/	/	/	/	/
Left lower lobe	/	/	/	/	Yes
Right upper lobe	Yes	/	/	/	/
Right middle lobe	/	Yes	Yes	/	/
Right lower lobe	/	Yes	/	Yes	/

## Discussion

In this retrospective study, we compared the clinical and pulmonary CT features of patients infected with the SARS-CoV-2 Omicron variant and the Alpha strain at a single tertiary center. Fever and cough were the most common symptoms in patients infected with Alpha strain, whereas “cold-like” symptoms characterized patients with the Omicron variant. Most patients infected with the Alpha strain had bilateral involvement and multiple lesions in the peripheral zones of the lung. In contrast, only some patients infected with the Omicron variant had mild, unilateral, and centrilobular foci. Most of the patients infected with the Omicron strain were asymptomatic, while patients infected with the Alpha strain were mainly the ordinary type. Our results suggest that the SARS-CoV-2 Alpha strain primarily affects the lungs, whereas the Omicron variant is restricted to the upper respiratory tract.

To the best of our knowledge, no previous study has compared clinical and pulmonary CT features of the patients infected with the Omicron variant and Alpha strain. Previous studies have reported that the five most common symptoms in the patients infected with Omicron were runny nose, headache, fatigue, sneezing, and sore throat (8). At the same time, fever, cough, and loss of sense of smell or taste were the most common symptoms in the patients infected with the Alpha variant.

Consistent with the above findings, the most common symptoms in patients with Omicron in our study were runny nose, sore throat, body aches, and headache. One study examined the clinical and epidemiological features of the first 40 patients with the Omicron variant in South Korea (10). It concluded that half of the patients were asymptomatic, while the remaining had mild symptoms. It is also noted that 6 of 40 patients showed pulmonary infiltrates on pulmonary images. Different with a study from China which reported the most common symptoms as fever, cough, and fatigue in 91 patients infected with the Alpha strain, “cold-like” symptoms were found to be the most common in patients with the Omicron variant in our study (17). More than 70% of 91 patients were symptomatic in their study, while 24.3% of 382 patients infected with Omicron were asymptomatic in our study (17). They also reported that 67.03% of patients with COVID-19 infected with the Alpha strain had bilateral pneumonia, as demonstrated by pulmonary CT images (17). The GGO in the pulmonary CT could help distinguish COVID-19 at an early stage of infection from community-acquired pneumonia (18). We analyzed data from 420 patients, including 382 with Omicron, and found that nearly a quarter of patients infected with Omicron but few patients infected with the Alpha strain were asymptomatic. Although there is a significant difference, we agree that the Omicron variant results in a higher proportion of asymptomatic-infected individuals than other variants of concern. Similarly, we found pulmonary infiltration in five patients in our study. Pulmonary infiltration on the pulmonary CT rarely occurs in patients infected with the Omicron variant because the Omicron variant was limited to the upper respiratory tract, with less involvement of the lungs. We also confirmed that the Omicron variant may cause less severe disease than the Alpha strain.

A study by researchers at the University of Hong Kong found that the Omicron variant infects the human bronchus more than 70 times faster and replicates faster than the Alpha strain. In contrast, the progenitor strain infects the lung more than 10 times quicker and replicates more rapidly than the Omicron variant (19). Hence, the clinical course is less severe, with fewer CT foci in the lungs but more symptoms in the upper respiratory tract in patients with the Omicron variant. This may also explain why the Omicron variant is transmitted from person to person more rapidly than the Alpha strain. However, we should note that the severity of COVID-19 in humans is determined by both viral replication and the host immune response to infection, which may lead to the dysregulation of the innate immune system (20).

Our study had several limitations. First, it was a retrospective study conducted at a single tertiary center. Second, the sample size of our study was small. Especially, the sample size of people infected with the Alpha virus was 10-fold smaller. Third, the patients infected with Omicron in our research were transferred from the isolation ward; therefore, most were young, asymptomatic, or with mild symptoms and without high-risk conditions seen in severe or critically ill patients. Some patients might be actually infected with the Alpha strain although 382 patients were presented at the hospital when the Omicron variant was dominant. Therefore, there was a possibility of enrollment bias. Fourth, the comparisons between 38 and 380 patients may be biased because there is a 10-fold difference between the numbers of patients in the two groups. Notably, 38 patients might be poor representative due to the small sample. Further studies with clinical data from more representative patients are needed to determine how the newest variant differs from previous variants of concern and its impact on the current COVID-19 pandemic.

## Data Availability Statement

The raw data supporting the conclusions of this article will be made available by the authors, without undue reservation.

## Ethics Statement

The studies involving human participants were reviewed and approved by the Medical Ethical Committees of Ningbo First Hospital (approval number: 2020-R05 and 2022RS065). Written informed consent for participation was not provided by the participants' legal guardians/next of kin because the requirement for written informed consents was waived since no additional interventions and potential harm were posed to these patients.

## Author Contributions

GQ and GC contributed to the conception and design of the study. NY, JD, and JY organized the database. CW and JiaH performed the statistical analysis. NY wrote the first draft of the manuscript. YF and JinH wrote the sections of the manuscript. DX was in charge of the critical revision of the manuscript. All authors contributed to manuscript revision, read, and approved the submitted version.

## Funding

This research was supported by the First Batch of Young Technical Backbone Talents Project of the Ningbo Municipal Health Commission (to NY), the TianQing Liver Diseases Research Fund Subject of the Chinese Foundation for Hepatitis Prevention and Control (No: TQGB20180358), the Subject Funding for the Department of Infectious Diseases (No: 2020001), the Natural Science Foundation of Zhejiang Province (No: Q17H010001), and the Key Program of the Natural Science Foundation of Ningbo (No: 202003N4019).

## Conflict of Interest

The authors declare that the research was conducted in the absence of any commercial or financial relationships that could be construed as a potential conflict of interest.

## Publisher's Note

All claims expressed in this article are solely those of the authors and do not necessarily represent those of their affiliated organizations, or those of the publisher, the editors and the reviewers. Any product that may be evaluated in this article, or claim that may be made by its manufacturer, is not guaranteed or endorsed by the publisher.
